# Supplementary material for: A service evaluation and stakeholder perspectives of an innovative digital minor illness referral service from NHS 111 to community pharmacy
Source: PLoS One. 2020 Mar 19;15(3):e0230343. doi: 10.1371/journal.pone.0230343 (PMC7082053; doi:10.1371/journal.pone.0230343)
Supplement: S1 Table — (DOCX) [file pone.0230343.s001.docx]

| **Item** | **Details** |
| --- | --- |
| **Brief name** | Digital Minor Illness Referral Service |
| **Why** | Urgent and emergency care receive and manage a significant patient burden. Some of these patients are enter this healthcare sector via NHS 111, and some of these patients may be suffering from minor ailments/illnesses or conditions. The service aims to refer patients for such low acuity conditions to community pharmacy for advice and management, thereby reducing pressure in higher acuity settings, but also to educate patients and the public about the capacity and capability available in community pharmacies. |
| **What** | A service specification was developed and shared with NHS 111, community pharmacy and service managers and commissioners. This was informed from stakeholder workshops to ensure participatory design. Training was provided to NHS 111 call handlers by project managers to educate about the services and products available in community pharmacy and rationalise the redirection away from urgent and emergency care. A web-based platform (PharmOutcomes) was utilised to aid in NHS 111 referral to community pharmacy and community pharmacy data entry about service delivery and patient outcomes. |
| **Who provided** | NHS 111 call handlers were provided with specific training about the service and the computer decision software used to triage/answer calls was adapted to ensure community pharmacy was profiled as a healthcare provider for certain minor conditions.  Community pharmacists were not provided with specific training, but were made aware of the service. Community pharmacists have knowledge and expertise in the differential diagnosis, treatment and appropriate self-care advice for minor conditions. |
| **How** | NHS 111 call handlers managed patient queries through the NHS 111 helpline. Referrals to community pharmacy were made electronically.  Community pharmacists either engaged with referred patients face-to-face in the pharmacy on presentation, or on the phone if the patient called the pharmacy or if the community pharmacist proactively contacted the patient. |
| **Where** | The PharmOutcomes platform enabled transmission of referred patient details to the community pharmacy. Community pharmacists could check their dashboard for referrals and then contact patients by phone, or they could wait until the patient presented at the pharmacy to manage the query and presenting condition. |
| **When and How much** | The service was started in December 2017 as a pilot in the North East region of the UK. The end date was extended past March 2018. In September 2018, the service was spread to other regions in England, and by October 2019 it was rolled out for national adoption. In the North East, approximately 1000 calls per month were being referred to community pharmacy. |
| **Tailoring** | The service was not adapted significantly since the initial implementation and subsequent wider adoption. Now there is some interest to investigate if more conditions could be referred to community pharmacy and supported with the establishing of patient group directions, e.g. supply of antibiotics for infections. There is also interest to expand the service so that referrals to community pharmacy can be generated by reception staff in General Practice. |
| **Modifications** | There were no modifications to the intervention during the period of evaluation reported in this study. |
| **How well** | The fidelity of intervention delivery was not assessed. However, interviews with NHS 111 call handlers demonstrate that if a patient refuses a referral to community pharmacy or if a call handler lacks confidence in referring to pharmacy, a patient could still be directed to urgent and emergency care for a condition that is flagged as appropriate for management within community pharmacy. Interviews with community pharmacists demonstrate that there is a variability in confidence and perceived capability and capacity to manage referred patients. This may contribute to unactioned referrals or escalations of patients back into NHS 111 or into urgent and emergency care. |
