# Supplementary material for: A service evaluation and stakeholder perspectives of an innovative digital minor illness referral service from NHS 111 to community pharmacy
Source: PLoS One. 2020 Mar 19;15(3):e0230343. doi: 10.1371/journal.pone.0230343 (PMC7082053; doi:10.1371/journal.pone.0230343)
Supplement: S2 File — (DOCX) [file pone.0230343.s003.docx]

**Community pharmacist topic guide**

**Introduction**

Firstly, I would like to audio record our interview so I can go over it later and type it up. I promise to destroy the recording after I have completed this study; would that be okay?

I am Hamde Nazar interviewing respondent number…………………………….

Thank you for agreeing to participate in this study which aims to investigate the opinions and experiences of community pharmacists providing care and advice for minor conditions to patients and the public through a new service directing patients from NHS 111 to community pharmacy. I would be grateful if you could give me a verbal confirmation that you have seen and read the information sheet and that you have read and signed the consent form/provide oral consent to undertake this interview.

This interview will take about 30 minutes to one hour. I am interested to understand your views and perspectives on the service being provided to patient and the public when they call NHS 111 for help with a minor condition. I would like to understand what you think about this service and if you think it is of value or if you have any suggestions for improvement. All information is confidential. It will not be possible to link the information you disclose back to you. Participating in this interview is entirely voluntary and your response will not your employment or .

I may ask you for clarification about what you describe and may ask for examples. In addition feel free to stop me if you have a question or need any clarification.

I would like to audio record our interview so I can go over it later and type it up. I promise to destroy the recording after I have completed this study; would that be okay?

So is it OK if we begin?

[the following are a list of questions that show the topics to cover and will be used to facilitate a conversational flow]

Qualitative interview questions for service providers

1. Are you registered to provide DMIRS?
2. Are you able to provide your initial thoughts about this service?

If participants provide this service:

1. When did you register for DMIRS and why?
2. What has been your experience of DMIRS since registering?
3. What is the process of a referral being received from NHS 111 to completion of the consultaiton? What are your thoughts on that?
4. What are your experiences with communications between you and other people involved in the referral process?
5. What are your experiences of using the NHSMail and PharmOutcomes [different regions may be using different platforms to receive referrals] or other communication platform?
6. How does DMIRS fit in to your daily workflow? What changes have you made to accommodate providing this service?
7. How long are the consultations normally? Can you take me through the steps you follow to complete a referral indicating how long each step might take?
8. If you speak to a patient before they come into your pharmacy, how is that and how long do those calls take?
9. How have patients responded to the service? Do you have any examples of cases (positive or negative) that would help us understand your and the patient’s flow through the service?
10. This is quite a general question but can I ask you to explain what role do you see for community pharmacists? Does DMIRS positively or negatively engage with that role?
11. Are there any barriers to providing this service in your pharmacy?
12. Are there any facilitators to providing this service in your pharmacy?
13. Are there any unintended consequences (positive and negative) that you know about of introducing DMIRS?
14. Is there anything within your pharmacy that you do in house to aid in the delivery of the service?

If participants do not provide this service:

1. Why did you not register to provide DMIRS?
2. What would encourage/enable to register to provide this service?
3. What would prevent/hinder you to register to provide this service?
4. Can you anticipate any benefits to providing this service, to you as the service provider and to the patients?
5. Can you anticipate any disadvantages to providing this service?
6. How do you think patients with minor conditions calling through to NHS 111 should be managed?
7. Do you think community pharmacists have a role to play in providing care and advice on minor conditions?

[Conclusion]

I am very grateful for giving me your time to tell me about your views and experience. It has really helped understand the perspective of the service providers. Before we finish this conversation, do think you have anything else to say about the service, or the care and advice that has or could be provided? Thank you very much for making time for this interview.

We would like to remind you that you have the contact details of the independent researcher on the participant information sheet if you would like to contact them for further information, clarification or with any concerns.

Also on the participant information sheet is the contact for someone outside of the research team who would be able to receive and manage any concerns or issues with the research and its conduct. Would you like either of these contact details again?

Again many thanks for your time and support for this study.

**Project team member’s topic guide**

**Introduction**

I am Hamde Nazar interviewing respondent number…………………………….

Thank you for agreeing to participate in this study which aims to investigate the opinions and experiences and thoughts of CPRS/DMIRS project team members. I would be grateful if you could give me a verbal confirmation that you have seen and read the information sheet and that you provide oral consent to partake in this interview and that it is audio-recorded.

This interview will take about 30 minutes to one hour. I am interested to understand your views and perspectives on how the service delivery is going. I would like to understand what you think about this service and if you think there are any particular barriers or facilitators. All information is confidential. It will not be possible to link the information you disclose back to you. Participating in this interview is entirely voluntary.

I may ask you for clarification about what you describe and may ask for examples. In addition feel free to stop me if you have a question or need any clarification.

I would like to tape record our interview so I can go over it later and type it up. I promise to destroy the tape after I have completed this study; would that be okay?

So is it OK if we begin?

[the following are a list of questions that show the topics to cover and will be used to facilitate a conversational flow]

Can you describe your role(s) on a day-to-day basis?

And how does that relate to the DMIRS/CPRS? How are you involved, and what is your contribution?

Can you tell me your thoughts on CPRS as a service? Do you think there was a need for this and why?

Can you tell me about your thoughts of the role of community pharmacy in the provision of healthcare? Are you able to describe how these perceptions have developed/changed over time?

How do you think the CPRS is going so far in the region? How do you think this compares to other regions?

How do you think any learning from the NE pilot has helped or hindered CPRS in your region?

How do you think the learning from the NE pilot was managed and disseminated to the other regions?

How do you think the service delivery in this region compares to the delivery in the NE?

Are you able to describe how the service is project managed in the area?

What are your thoughts on how this management is going?

What are the factors that contribute to the success of the service?

Do you think there are any factors particular to your region that have helped the CPRS delivery?

What are the factors that act as barriers to the service?

Do you think there are any factors particular region that are hindering the delivery of CPRS?

Are you able to comment on how the design and delivery of the CPRS compare to other services that you have also been involved in or had experience with?

Are you able to describe your involvement in any engagement work undertaken in the region? How do you think this went?

Are you able to comment on the resources, marketing, training that was invested in CPRS in the region?

And are you able to comment on the funding model in place for CPRS? What are your thoughts on how this service be sustained?

What do you think are the risks to the sustainability of this service?

What are your thoughts on the capacity within community pharmacy for CPRS?

How do you think this service fits, or not, with the recent NHS long term plan?

[Conclusion]

I am very grateful for giving me your time to tell me about your views and experience. Before we finish this conversation, do think you have anything else to say about the service? Thank you very much for making time for this interview.

We would like to remind you that you have the contact details of the independent researcher on the participant information sheet if you would like to contact them for further information, clarification or with any concerns.

Also on the participant information sheet is the contact for someone outside of the research team who would be able to receive and manage any concerns or issues with the research and its conduct. Would you like either of these contact details again?

Again many thanks for your time and support for this study.

**Patient and Public topic guide**

**Introduction**

I am Hamde Nazar interviewing respondent number…………………………….

Thank you for agreeing to participate in this study which aims to investigate the experiences of care and advice for minor conditions received by patients and the public through a new service directing patients from NHS 111 to community pharmacy. I would be grateful if you could give me a verbal confirmation that you have seen and read the information sheet and that you have read and signed the consent form.

This interview will take about 30 minutes to one hour. I am interested to understand your views and perspective on the service you received when you called NHS 111 for help with a minor condition. I would like to understand if you found the service helpful or if you have any suggestions for improvement. All information is confidential. It will not be possible to link the information you disclose back to you. Participating in this interview is entirely voluntary and your response will not affect any other healthcare you access in anyway.

I may ask you for clarification about what you describe and may ask for examples. In addition feel free to stop me if you have a question or need any clarification.

I would like to tape record our interview so I can go over it later and type it up. I promise to destroy the tape after I have completed this study; would that be okay?

So is it OK if we begin?

[the following are a list of questions that show the topics to cover and will be used to facilitate a conversational flow]

So can you tell me about when you called NHS 111 for a minor condition?

Why did you call them?

What prompted you to call them?

How serious did you think the condition was/how urgent did you think the need for help was? Had you called them for something similar in the past and what had happened?

What was your expectation for advice?

How did the call go?

Did you feel listened to?

Did you feel you were able to provide enough information about what was wrong?

How did the call handler recommend what to do next?

How did this make you feel and why?

Did you want to follow their advice?

Did you follow their advice and why?

What happened next?

(if they didn’t go to the pharmacy)

Why did you choose not to go to the pharmacy?

What are your thoughts of the advice and care you can get from a community pharmacy?

How would you feel going to a community pharmacy for help for such a condition and why?

Is there anything that gives you this impression?

Is there anything that might change your opinion?

So where did you go/what did you do to help with this condition?

And how did this plan work out and why?

What did you think of the overall experience?

What was really good about the experience?

What was not good about the experience?

If you had the condition again, what would you do?

(if they went to the pharmacy)

How did you find going to the community pharmacy?

How did you find talking to the pharmacist?

How did you find the advice they provided you?

How did you find the treatment they provided you?

How did you find you managed your condition after that?

Did you have to seek help from anyone else and why?

What did you think of the overall experience?

What was really good about the experience?

What was not good about the experience?

If you had the condition again, what would you do?

What are your perceptions of what a community pharmacist can do about minor conditions?

Is this different to before the service?

[Conclusion]

I am very grateful for giving me your time to tell me about your views and experience. It has really helped understand what it might be like from the patient and public perspective. Before we finish this conversation, do think you have anything else to say about the service, or the care and advice you received? Thank you very much for making time for this interview.

We would like to remind you that you have the contact details of the independent researcher on the participant information sheet if you would like to contact them for further information, clarification or with any concerns.

Also on the participant information sheet is the contact for someone outside of the research team who would be able to receive and manage any concerns or issues with the research and its conduct. Would you like either of these contact details again?

Again many thanks for your time and support for this study.
